# BUILDING ACADEMIC CAPACITY IN PHYSICAL AND REHABILITATION MEDICINE. A Background Paper by a working group of the European Academy of Rehabilitation Medicine

**DOI:** 10.2340/jrm.v56.40468

**Published:** 2024-06-11

**Authors:** Bengt SJÖLUND, the working group

**Affiliations:** University of Southern Denmark, Deptartment of Public Health, Campusvej 55, DK-5230 Odense, Denmark

The reason for an action of the European Academy of Rehabilitation Medicine in the area of academic capacity is to develop the Field of Physical and Rehabilitation Medicine (PRM) based on scientific findings, both regarding biological mechanisms as well as functional assessments and therapies to provide effective rehabilitation to all Europeans in need thereof. Our academy has, with other European organizations, already established a Cochrane center, devoted to evidence-based rehabilitation in PRM.

One general problem in this area is that it may be in itself insufficiently mapped out as a scientific field. In medicine, it tends to be fragmented conceptually across many organ areas. Linking rehabilitation with physical medicine, and thus focusing attention away from psychological, social, educational and psychiatric contributions, may also be inadequate. One reason that few doctors do rehabilitation research may in fact indicate that other disciplines need to be integrally involved with PRM for the formulation of research questions. Therefore, a clear definition is deemed important to gain insight for and acceptance by our colleagues in other areas of medicine.

On the other hand, the paper by Stucki et al on rehabilitation sciences in the ICF context (1) advocates that research capacity in human functioning and rehabilitation research should be developed from the comprehensive perspective based on the ICF-model. Moreover, the rapidly developing field of neurobiology with plasticity, formation of adult stem cells and other long term changes in adult brain function may give PRM a unique basis in the concept of ‘Applied neurobiology’ that can be developed both from motor, sensory and cognitive neuroscience and also from psychology. ‘Applied neurobiology’ would focus on long term changes in nervous function as apart from emergency neurology. (See ‘The neurobiological background to rehabilitation’ (2))

Another problem identified is the lack of exposure to PRM in that students in medical schools are usually not aware of PRM as an independent specialty. They are not exposed to physicians, residents, or patients in PRM during their undergraduate period. Consequently they will not consider P&RM as a potential future career and choose another speciality. This point agrees with a problem acted upon in the action plan of the American Rehabilitation Medicine Summit (3): ‘Lack of exposure to rehabilitation and rehabilitation research’. They recommend specific undergraduate and graduate programs to expose other researchers to rehabilitation research and to extend research training.

The list of ‘underexposures’ is considerable and includes

- Lack of basic training in research during PRM training
- Lack of exposure to academic “culture” during PRM residency
- Lack of participation in research during PRM training
- Lack of signposting to those who could advise on the academic route
- Lack of continuing support for budding academics
- Lack of interest/possibilities of PRM doctors to work in academic department
- Lack of qualified candidates to become Professors in PRM
- Lack of interest of qualified persons to become Professors in PRM

We recommend that when medical students arrive in the phase when they (have to) decide on their future career and have to choose a specialty, they are already aware of the existence of PRM, the most common patient categories, the most common interventions and have a global understanding of functioning (ICF), the way PRM is organized in their country and what to do to be accepted in a residency program in P&RM. It should also be remembered that PRM physicians are the only rehabilitation professionals with a 4-5 year long clinical training under supervision after graduating, and including a multidisciplinary approach, before becoming independent PRM specialists.

Furthermore, university hospitals with an affiliated medical school should have an independent department of PRM with a full professor in PRM and his/her staff with teaching and education as (one of the) main responsibilities. Members of his/her staff should not only be judged on research output, but also on activities/qualifications in education and teaching. Besides teaching, the PRM department should also offer possibilities for students to participate in clinical and research activities (e g lectures, patient demonstrations and literature searches/seminars). Thus, PRM should be well represented in the undergraduate medical curriculum. For example, in one Finnish university (Oulu), the PRM & Rehab course will now be 5 weeks long, including bedside teaching and a multiprofessional approach. A problem solving approach in case analysis is recommended, as is to actively seek young medical students from the 1st and 2nd year courses for PhD students as then they may even have time to finish their thesis before they graduate to physicians. An advantage is then to have access to good clinical or epidemiological data sets for their work.

To solve the present situation, we recommend that, wherever possible,

- A certain share, e g 1%, of the clinical budget is ideally reserved for research projects
- Residents in PRM should always get 10% research time, at least in University Hospitals.
- Young coworkers should be involved in all research projects (and become coauthors).
- The quality of international PRM conferences/congresses should be continually improved.
- Public awareness about PRM is created, e g the cost saving effects of evidence based rehabilitation (cf 4).

The Academy could specifically

- create ‘Web Communities’of PRM PhD students and of Academic PRM specialists
- cooperate with organizations for the disabled on advocacy, e g by creating an Academy Liason to the European Disability Forum.
- consider to ask for support from responsible authorities in the EU countries or possibly from the EU commission in Brussels

If actions are not taken, there is a risk of de-academisation of PRM, occuring when a clinical department of PRM at a University Hospital is not chaired by a PRM specialist, but by a PhD or a non-PhD with a non-medical background, e g a physiotherapist, a psychologist or a movement scientist. Non-medical persons are less likely to address clinically relevant research questions in PRM and research might become less comprehensive and more monodisciplinary. As an end effect, Evidence-Based Medicine might even be replaced by opinion based medicine. Moreover, the quality of PRM teaching and training is likely to suffer if it becomes the responsibility of non-medical staff.

Related to this problem is the presence of many PhD students with a non-medical background as reported from several countries. They are cheaper to hire, often younger with less family burden and have sometimes had more research training than people with a MD background. They thus compete with MD PhD candidates who also have a money barrier to continue an academic career. On the other hand, it is important to include other rehabilitation profession categories among PhD candidates and researchers in the rehabilitation research groups, allowing work in multidisciplinary collaborative projects.

Nevertheless, we should demand via the UEMS/PRM Section that medical rehabilitation activities should always be under the direct directorship of an MD, at least in University Hospitals. A similar demand can be posed via the European commission in Brussels or individually to authorities in all EU countries. The possibility to cooperate with Organizations of the Disabled on this issue should be examined. There is also a responsibility for PRM professors to recruit a fair share of MDs as PhD students.

To maintain academic PRM doctors after dissertation to form a basis for recruitment of professors and to increase scientific productivity in PRM (even if there are large variations in possibilities between European countries) we recommend to

- Create an academic environment with part time academic positions, allotted time and space for research, allowing regular interaction also with academics doing other programmes.
- Develop supervision and mentoring
- Belong to an academic department (with a support structure and regular research meetings) even if working far away
- Keep the person involved in the department with functions on a regular basis
- Create access to role models - people with research and grant-getting ability should be accessible and there should be many opportunities to interact
- Develop prizes and then give publicity to them such as in scientific journals.
- Inform about funding streams, government - and other funding agencies
- Create a national academic group with the help of National PRM Societies which supports, provides peer group and professorial availability and policy, interacting with government, also with creating incentives to continue.
- Create awareness in public and supporting patient groups

We are aware that there are large variations in possibilities between European countries. In some countries, e g in the UK, there are initiatives for all clinical specialties, the academic clinical fellowships, to finance research opportunities and give support but these are subject to considerable competition. Similar fellowships exist in Sweden but only for residents. Introduction in other countries would probably take political decisions. One recent small survey in a southern European country indicates that at present, PRM specialists could only spend 3 hours/week on academic tasks and published less than one paper per year, mostly in national journals.

In summary, our recommendations on actions to be taken to improve academic capacity in PRM will involve European PRM leaders on all levels: locally, regionally and nationally. Visibility, structured mentoring and financing are key elements in this work that will eventually make rehabilitation more effective to benefit all those persons in need of personal empowerment.
